# Medicine Inventories and Practices of Self-Medication in Households of Medical and Pharmacy Students at the University of Rijeka, Croatia: A Cross-Sectional Study with Implications for Healthcare Professions Education

**DOI:** 10.3390/pharmacy14050116

**Published:** 2026-08-07

**Authors:** Vedrana Aljinović-Vučić, Jasenka Mršić-Pelčić

**Affiliations:** Department of Basic and Clinical Pharmacology and Toxicology, Faculty of Medicine, University of Rijeka, Braće Branchetta 20, 51000 Rijeka, Croatia; jasenka.mrsic.pelcic@uniri.hr

**Keywords:** self-medication, medicine storage, home pharmacy, medical students, pharmacy students, healthcare professions education

## Abstract

Background/Objectives: Self-medication and keeping medicine inventory in households are common practices, even among healthcare professionals and healthcare professions students. Despite their knowledge of appropriate medicine use and storage, these practices are not always responsible and may pose health risks. This study aimed to describe self-medication practices, contents of home pharmacies, and habits of keeping medicine inventories in households of medical and pharmacy students at the Faculty of Medicine, University of Rijeka. Methods: In this cross-sectional study conducted in 2025, 60 third-year medical and pharmacy students interviewed members of their households regarding contents of home pharmacies and self-medication practices and compiled inventories of medicines kept at home. Results: Of the participating students, 70.0% were female. Healthcare professionals (physicians or pharmacists) were present in 23.3% of households. Medicines were stored in a designated location (“home pharmacy”) in 65.0% of households, whereas only one household reported having no medicines. Expired medicines and/or medicines with an unknown purpose were found in 41.6% of households. No household reported that medicines were accessible to children younger than 14 years. Inventories of stored medicines were obtained from 54 households (90.0%). Analgesics and antipyretics were present in 92.6% of households and were used for self-medication in 96.0% of them. Cough and cold preparations were found in 35.2% of households and used for self-medication in 94.7%. Antibiotics were present in 31.5% of households and used for self-medication in 52.9%, while anxiolytics were found in 16.7% of households and used for self-medication in 33.3%. Conclusions: Stocking medicine supplies and practicing self-medication are common among households of third-year medical and pharmacy students at the Faculty of Medicine, University of Rijeka. Analgesics and antipyretics were the most frequently stored medicines used for self-medication. The presence of expired medicines and the self-medication of prescription-only medicines highlight the need for greater emphasis on responsible self-medication, safe medicine storage, and rational medicine use within healthcare professions education.

## 1. Introduction

Self-medication is a part of the broader term “self-care” defined by the WHO as a set of actions that an individual or a community undertakes to protect, maintain, and recover their own health [1]. In that group of activities, self-medication refers to taking drugs without the supervision of a healthcare professional (HCP) [2]. Self-medication can offer opportunities such as faster and more convenient solutions for a health issue. This can be beneficial for an individual and for decreasing the burden on overwhelmed health systems. However, for self-medication to be responsible and appropriate, people who engage in that practice must recognize an ailment themselves and choose an appropriate therapy without consultation with an HCP. Otherwise, risks can arise from misdiagnosis and wrong drug choice, such as disease progression caused by using the wrong drug or treating only symptoms while potentially more serious underlying conditions keep developing [3]. Medicines people use for self-medication can include those previously prescribed by physicians which patients later use independently, leftovers from previous treatments, or non-prescription medicines. Non-prescription medicines are intended to be used for self-medication because, due to their favorable risk–benefit ratio, they are available over the counter (OTC) and can be taken without HCP’s supervision when used as intended [2]. However, these drugs can have serious potential adverse effects and health dangers, especially when not used responsibly [4,5,6,7]. Self-medication with prescription-only medicines, such as antibiotics and anxiolytics, can pose even higher health risks to individuals using them irresponsibly and these practices can also increase risks such as development of antimicrobial resistance (AMR) in an entire community [8,9,10].

Today, self-medication is widely practiced in different populations. Although professional organizations such as the American Medical Association and the United Kingdom General Medical Council recommend that HCPs do not treat themselves, members of their families, or other persons with whom they have close personal relationships, published research has shown that HCPs often engage in self-medication practices with prescription and non-prescription drugs [11,12,13,14]. This also applies to the students of healthcare studies [10,15,16,17,18,19,20,21]. Although HCPs are well educated in pharmacology and on correct use of medications, the previous literature has shown that HCPs do not always practice responsible self-medication [14,22]. This finding applies equally to practicing HCPs and to students of healthcare professions who are yet to assume HCP roles in the future [10,13]. This observation is important from at least two aspects: as HCPs or future HCPs who treat or will treat patients in the future and as users of medicines themselves. Irresponsible self-medication practices in HCPs and healthcare professions students reflect the need to reinforce knowledge on this topic in healthcare professionals’ education. According to research findings, HCPs often find practicing self-medication more convenient than consulting another HCP and thus keep using drugs without another healthcare professional’s advice or supervision while in some cases, due to the nature of the disease, e.g., psychiatric disorders, they avoid consultation with other HCPs due to embarrassment and fear of stigma and the influence it might have on their reputation [23,24,25]. These findings show a discrepancy between gained knowledge and personal practices.

Another parameter that should be addressed in healthcare professions education is hoarding medicines at home and related practices. Keeping medicine supplies at home can pose the risk of accidental drug poisoning, especially with drugs of unknown purpose or if more sensitive populations, such as young children, have access to these drugs. Additionally, it has been shown that storing medicines at home may lead to their greater utilization since it allows for self-medication even with prescription drugs [18,26,27]. Previous research has shown that hoarding medicines at home is commonly practiced for various reasons, such as changes in medicine, oversized packages, and for future use or reuse [28,29,30,31,32,33,34]. Hoarding medicines is often irresponsible as they are often stored without packages, expired, or present in abundance [31,32,33,35]. Additionally, it was reported that medicines kept at home were available to children younger than 6 years of age, which increases the risk of possible poisoning [30]. Analgesics and antipyretics were frequently found in home medicine cabinets, in addition to antibiotics and psychotropic medicines, and both groups of prescription-only drugs were used for self-medication [30,32,33,34,36]. Tourinho et al. reported that significantly larger supplies of analgesics–antipyretics, and antibiotics were found in the homes of patients who practiced self-medication [30]. Additionally, in a study involving medical students, Hu et al. showed a high level of self-medication with antibiotics in parallel with keeping supplies of antibiotics at home [37]. Hence, it is important to gain insights into the habits of keeping medicine supplies in different populations and to increase awareness of both their safe storage and self-medication practices.

This study aimed to investigate the contents of medicine cabinets in the households of students in medicine and pharmacy, and to describe self-medication practices and frequently used medicines in those households. As future healthcare professionals who are gaining a thorough education on medicines and their proper use, they are expected to develop and show above-average knowledge on responsible drug use in their households. Insights into this field can reveal implications for educational needs for healthcare professions students in the aspect of medicine hoarding and self-medication; this study may also inform future decisions about possible needed interventions in the current curricula of healthcare professions studies.

## 2. Materials and Methods

### 2.1. Study Design and Setting

The survey was performed at the Faculty of Medicine, University of Rijeka, among third-year medicine and pharmacy students who were enrolled in the pharmacology course in 2025. Third-year students received written information on the purpose of the study and its voluntary and anonymous nature, along with its objectives and the type of data being collected. Questionnaires were distributed to students in person during pharmacology courses, and students were instructed on how to complete them. Information sought referred to the students’ households. Students were instructed on how to conduct structured interviews with their household members, how to inspect their family household medicine supplies, and how to fill in the questionnaire concerning self-medication practices in their households. Participants were informed that they can withdraw at any time without any consequences. Those who consented proceeded to complete the questionnaire. This study was conducted in accordance with relevant institutional and ethical standards, as well as with the 1964 Helsinki declaration and its later amendments or comparable standards.

### 2.2. Study Instrument

The study instrument was the questionnaire, which had already been used in previous research on self-medication and household drug inventories of medicine and pharmacy students. It was developed by the authors of previous studies, and an identical methodology was used in our survey [32,33]. The questionnaire comprised multiple choice questions which collected data on the following: (1) the type of study (medicine or pharmacy); (2) gender; (3) presence of healthcare professionals educated on appropriate medicines use in the household (medical doctor; doctor of dental medicine; doctor of veterinary medicine, pharmacist); (4) predominant/main source of advice on drug use (independent HCPs—no family member; HCPs in household; product information leaflet in a drug package; friends/family—no HCPs); (5) the manner in which medicines are stored in the household (presence of a designated area for keeping medicines, such as “home pharmacy”/”medicine cabinet”, or whether they are scattered around the household); (6) availability of drugs to children younger than 14 years (“yes”, “no”, or “no children in the household”); (7) characteristics of household drugs inventories (presence of expired drugs, drugs of unknown purpose, or both); and (8) a standardized table recording data on drugs found in households (for each drug found, students recorded the trade name, generic name, unit strength, package size, number of packages, expiry date, whether the purpose or indication of the drug was known, and information on how often each drug was used for self-medication without supervision or recommendation of HCPs, with possible answers being “regularly”, “sometimes”, and “never”). Self-medication was defined as the use of medicines without consultation with an HCP on their purpose/indication, appropriate dosage, or duration of treatment. It included the following possibilities: (1) a person decides on medicine use completely independently or (2) a medicine was previously prescribed by a physician, but the person changes its dosage or treatment duration, stops the treatment and later restarts it, or uses that specific medicine to treat another ailment on their own will. The data collected were checked by a pharmacist and a physician, and individual drugs were pooled into categories based on pharmacotherapeutic groups found in each household. The survey instrument is presented Supplementary Materials.

### 2.3. Statistical Analysis

The study was descriptive and observational in its nature, with no inference intended. Hence, no formal statistical analysis was performed. Data were analyzed using descriptive statistics, and the results are shown as percentages and distribution of frequencies.

## 3. Results

### 3.1. General Characteristics of Participants and Households

Out of the 160 students invited to participate, 60 accepted and returned valid questionnaires, with a response rate of 37.5%. Over two-thirds of participants were female (70.0%). Healthcare professionals (physicians, pharmacists) were present in 14 (23.3%) households. Children younger than 14 years of age were present in 31.7% of households. The main characteristics of the participants and households are shown in Table 1.

### 3.2. Medicine Storage Habits in Surveyed Households

Medicines were found in 98.3% of households, and only one household had no medicines at all. In nearly two-thirds (65.0%) of the surveyed households, drugs were kept at a specific place in the apartment (“home pharmacy”), while they were scattered around the house or apartment in 33.3%. Drugs with past expiry dates and/or of unknown purposes were found in 41.6% of households, while no such drugs were reported in 56.7%. Children younger than 14 years were present in 31.7% of households, and drugs were not available to them. In total, 68.3% of households had no children < 14 years. The main information about drug storage in the surveyed households is shown in Table 2.

Valid medicine inventories were provided by 54 households. Analgesics–antipyretics were found in most households for which a medicine inventory was provided (92.6%). They were followed with cough and cold products (35.2%), antibiotics–chemotherapeutics (31.5%), and anxiolytics (16.7%). A variety of other medicinal products belonging to different pharmacotherapeutic groups was found in 31.5% of households surveyed. The list of pharmacotherapeutic groups of drugs found in the surveyed household is shown in Table 3.

### 3.3. Practices of Self-Medication in Surveyed Households

**Figure 1:**
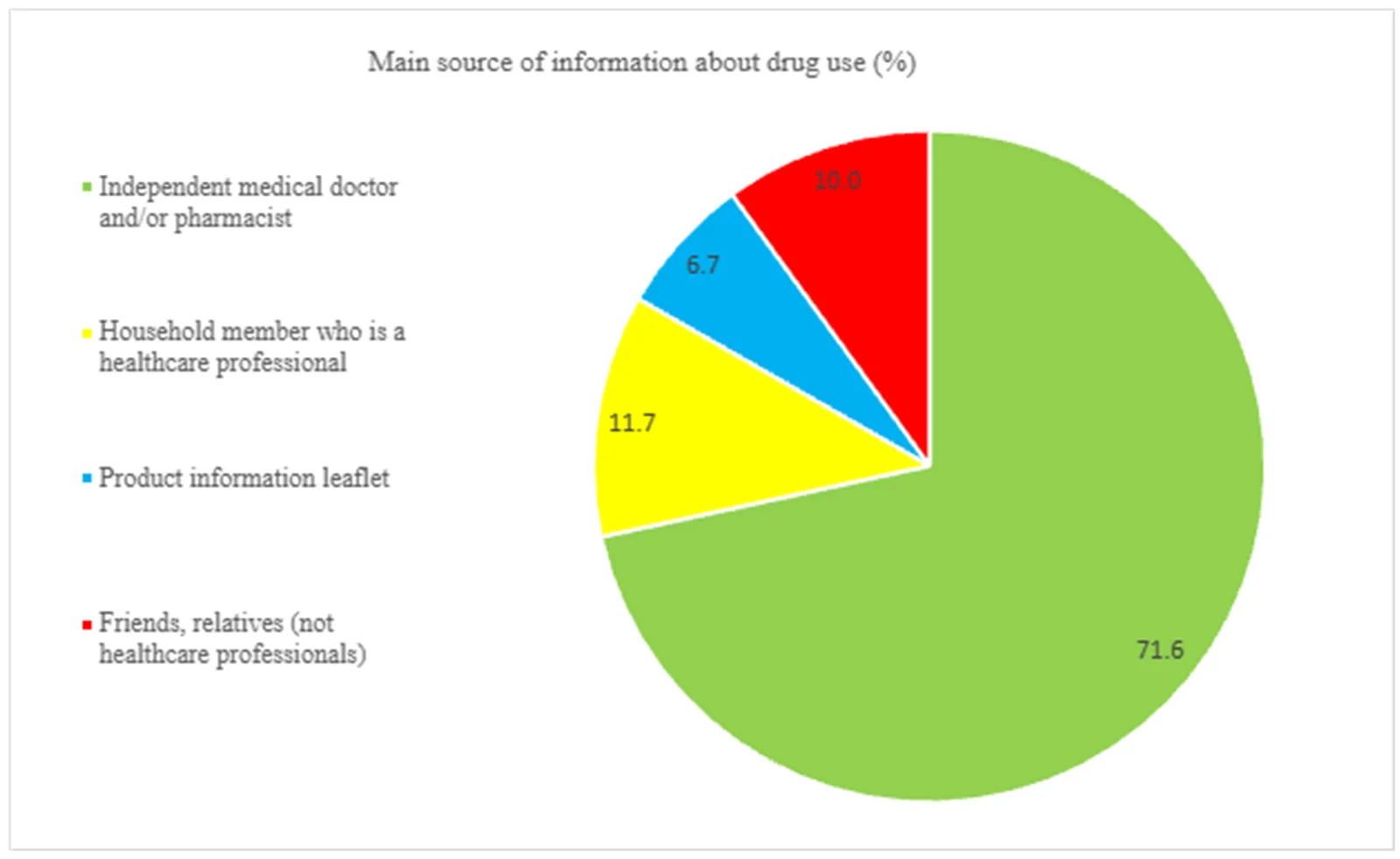
Main source of information about drug use (valid N = 60).

The main/predominant source of information about drug use was an independent physician and/or pharmacist in 71.6% of households surveyed, followed by a household member who was a healthcare professional (11.7%) and a product information leaflet (6.7%). Friends and/or relatives who were not HCPs were disclosed as the main source of information about drug use in 10.0% of households surveyed. The breakdown of the main sources of information about drug use is shown in Figure 1.

**Figure 2:**
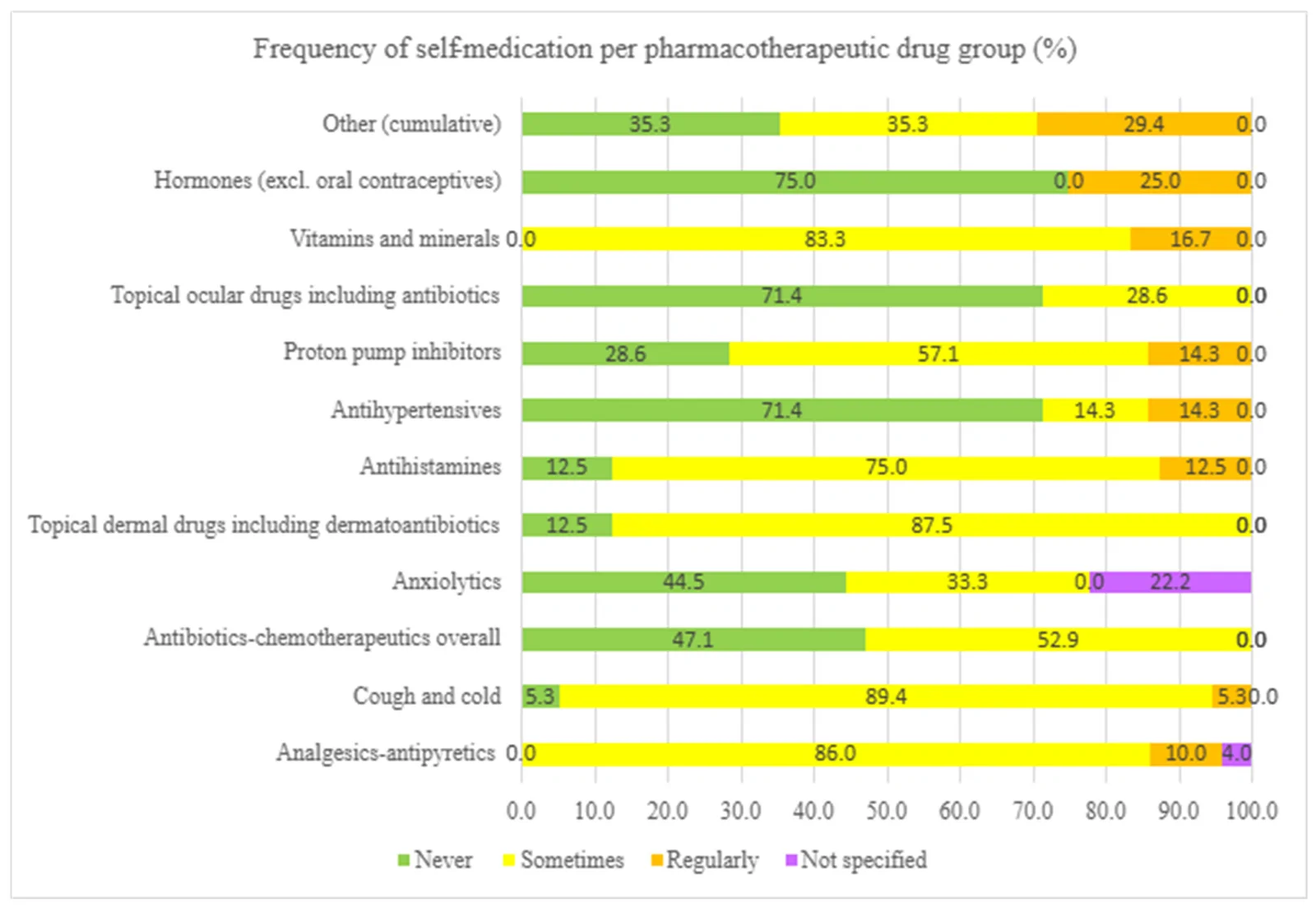
Frequency of self-medication based on pharmacotherapeutic groups of drugs (percentages were calculated based on the number of respondents who answered the question for each pharmacotherapeutic group).

Self-medication with analgesics–antipyretics was reported in 96% of households in which they were found, followed by cough and cold products in 94.7%. Other groups of drugs for which a rather high level of self-medication was disclosed were topical dermal drugs (87.5%), antihistamines (87.5%), and proton-pump inhibitors (PPIs) (71.4%). Considering prescription-only drugs, self-medication with antibiotics–chemotherapeutics was disclosed in more than half of the households in which they were found (52.9%). Use of anxiolytics for self-medication was disclosed in 1/3 of households in which they were found (33.3%), followed by antihypertensives (28.6%) and hormones (excluding oral contraceptives) (25.0%). The frequency of self-medication based on pharmacotherapeutic groups of drugs found in surveyed households is shown in Figure 2.

## 4. Discussion

This study explored the contents of home pharmacies and identified medicines pertaining to more than fifteen different pharmacotherapeutic categories. Medicines stored in most households were analgesics–antipyretics, which is expected based on other published studies [15,17,32,33]. In part, these medicines can be obtained without a prescription, which contributes to their accessibility and presents a probable reason for their presence in almost all households. Notably, although these medicines can be obtained without a prescription and can generally be used by patients to treat minor ailments as intended, these medicines can pose risks to patients’ health if used inappropriately or in large quantities. They can be especially dangerous if available to children in quantities which could be harmful for them. A reassuring finding was that neither analgesics–antipyretics nor other groups of medicines were accessible to children < 14 years. We also found that prescription-only pharmacotherapeutic groups of medicines such as antibiotics, anxiolytics, antihypertensives, and hormones were stored in homes. These medicines are not intended to be used for self-medication. Nevertheless, practicing self-medication with them was disclosed. Storing drug supplies in households has been previously shown to facilitate self-medication [18,26,27]. Wang et al. disclosed this as a facilitator of self-medication with antibiotics, and it was shown that students who were storing antibiotics at home were five times more likely to practice self-medication with them [27]. In addition, it was described that self-medication was practiced in households which kept medicine supplies [30,37]. This is a feature worth consideration because investigating the contents of home medicine cabinets can reveal findings relevant in planning future interventions. These interventions should aim to increase awareness of the possible risks, such as poisoning, associated with keeping expired or unlabeled drugs at home. Notably, drugs with a past expiry date and/or of unknown purpose were found in 41.6% of surveyed households, which confirms the need to increase investigations of at-home drug supplies. HCPs and healthcare professions students are expected to have above-average knowledge of responsible drug use. However, the results show that their knowledge is often not reflected in their behaviors. The results of previously published research indicate that education did not translate to practicing responsible self-medication and that recognizing the risks associated with inappropriate medication use did not prevent healthcare professionals and students of healthcare professions studies (medicine, pharmacy, and nursing) from engaging in such practices [15,18,32,33,38,39,40]. Furthermore, Benameur et al. showed that 79.9% of healthcare sciences students were aware that self-medication with antibiotics was inappropriate, yet 58.4% reported using antibiotics for self-medication, showing discordance between their knowledge and behavior [41]. In our study, antibiotics were found in approximately one-third of households surveyed, which could be considered rather high given that these are prescription-only medicines intended to be used at the time of prescription instead of being stored. It could be speculated that the stored antibiotics were procured for personal use. This might also be the case for other prescription-only medicines that were stored; however, further research is warranted. Also, this could confirm the intention of self-medication with these medicines, and in the case of healthcare professions students, it could show that their awareness on appropriate medicine use needs to be addressed earlier in their education.

The results of this study show that self-medication is a prevalent practice in the surveyed households of medicine and pharmacy students. Analgesics–antipyretics were at the top of the list of drugs used for self-medication, which is in accordance with the results of other published studies [15,17,32,33,40]. Some of these medicines can also be obtained without a prescription and are intended to be used for self-medication for management of minor ailments. These were followed by cough and cold medications, topical dermal drugs, antihistamines, and proton-pump inhibitors, which are also medicines that can be obtained without a prescription but can also be prescribed by a physician. However, regardless of how they were obtained, studies show that the general population and HCPs use them on their own, and self-medication with these groups of drugs can be high and not always in line with the guidelines [13,42]. Moreover, self-medication with prescription-only drugs, such as antibiotics and anxiolytics, was found to be rather high: this was disclosed in 52.9% and 33.3% of households in which they were found, respectively. These findings raise concerns, especially considering that these medicines are prescription-only drugs that are not to be used without a physician’s recommendation. Similar studies have shown that HCPs and healthcare students practice self-medication with these drugs [17,21,25,32,33,37,41]. It is important to note that the practice of physicians treating themselves or close family members is not forbidden, though the rules are stricter for controlled drugs, opioids, psychotropic drugs, and other medicines which can cause development of dependence. Although the regulations on physicians prescribing medicines for themselves or close family members differ among European countries, this practice is generally not recommended [43,44,45]. A matter of concern in these situations is maintaining objectivity and professional distance. This practice may occasionally lead to unnecessary use of some medicines, such as antibiotics, which is caused not due to a lack of knowledge but to higher access to medicines. This has already been shown in previous publications revealing that the existing knowledge of HCPs or healthcare professions students were in discordance with their practices [15,39,42]. These observations highlight the need for planning interventions to increase awareness of appropriate self-medication earlier in the education of healthcare professionals.

The findings show that for most households, the main source of information about proper drug use was physicians or pharmacists, either independent ones or those living within the household. These could be viewed as encouraging findings, which may reflect a high level of confidence in the advice of educated healthcare professionals among surveyed households and present an opportunity to influence the knowledge and practices of patients in general. However, 10% of households disclosed that their primary source of advice on proper drug use was friends/relatives who were not healthcare professionals. This shows that even more effort is needed to minimize the habit of taking advice from non-HCPs on appropriate use of medications.

The study’s findings show that there is a need to intervene in education at universities and to develop more engaged courses so that students of medicine and pharmacy have better knowledge of proper self-medication and medicine storing practices. Another important aspect of surveying healthcare students’ practices is not only showing their personal preference but also showing indicators of behaviors they will express in their future roles as HCPs. This should be achieved not only by transfer of knowledge but also by developing critical thinking and recognizing their responsibility as professionals in developing appropriate self-medication practices. The findings of this study point to a need for educational initiatives at the level of higher educational institutions. They should be directed towards not only understanding the mechanism of action of drugs and which diseases they treat, but also for whom and when it is acceptable to use them, as well as when and how to engage in responsible self-medication. Ethically, it is also important to internalize healthcare students’ perception of their roles as future HCPs who will be providing professional advice about drug use to their patients and who should also be good examples of practicing responsible self-medication. Appropriate education, which increases awareness of responsible self-medication practices, should start with university institutions’ interventions. They should be included early in the curricula of healthcare professions studies so that students can learn to act responsibly and present exemplary behavior in the future. Besides practical knowledge on drug mechanisms of action and their targeted use, it is important to develop professional competencies and promote responsible practices. These features should be built up over the years of healthcare professions education, either through different parts of the curricula which are connected to pharmacology, public health, and professional ethics, or through introducing dedicated syllabi committed to responsible utilization of medicines and self-medication, which would integrate knowledge and faculty from different specialties. For these interventions to be successful, practical simulations and case studies should be developed and introduced, such as through the implementation of digital education, including video materials, digital applications, and simulations. These would be particularly useful in enabling students not just to hear and memorize information but also to experience training in real-life situations. Creating scientifically based solutions that rely on digital technologies and are accessible to students as tools for learning and for training could improve education and could also be used later in clinical practice.

## 5. Limitations

This study has certain limitations. First, it was designed as a cross-sectional study, meaning the findings should be viewed as correlations rather than causations. The aim was to provide descriptive insights into future healthcare professionals’ behavior with no inference intended; hence, it is important to note that the results should not be generalized. Also, the sample was not representative of the population of surveyed students, nor was it intended to be representative of this or any other population (such as “university students” or “medical and pharmacy students” or the “Croatian population”). The study mainly aimed to observe the current situation of appropriate drug use in a selected group of students who are expected to care about the appropriate use of drugs and to gain insights into the status of this issue at an institution where this kind of survey was not conducted previously. The aim was not to discover facilitators of self-medication. Hence, formal questionnaire validation, pilot study, and reliability testing were not conducted.

The study did not investigate medicine storage conditions such as temperature or whether they were kept away from light, nor the exact number of medicines stored without their original packages or a product information leaflet. In future research, these parameters should be investigated to obtain more informed insights into real situations regarding medicine storage in households.

Nevertheless, it should be acknowledged that although the surveyed population was not large, the results of our study are comparable to similar studies previously performed both in Croatia and internationally. As such, this study can contribute to delivering a feasible solution. Although the study was cross-sectional, it used the same methodology as in previously published studies and revealed comparable findings that self-medication practices, as well as medicine storing habits, are persistent and ingrained in populations of surveyed medical and pharmacy students.

## 6. Conclusions

The findings of this study on self-medication practices in households of medical and pharmacy students, together with findings from inspection of household medicine inventories, show that there is a need to address responsible self-medication early in healthcare professions education. Hence, it is important to develop curricula that will aim to encourage more responsible self-medication behaviors and to enhance the competence for recognizing self-medication patterns in patients and managing them. This also applies to the habits of hoarding medicine supplies at home, since this has been implicated as a facilitator of self-medication with different drugs, including antibiotics. There is a need to act earlier to increase awareness of responsible self-medication and storing medicine at home. Additionally, students’ perception as responsible role models should be developed from the beginning of their education.

Medical, pharmacy, and other healthcare professions students need to be guided and supervised in this aspect to internalize positive practices and to preclude the adoption of irresponsible behaviors. Eventually, research on this topic could contribute to the development of generations of healthcare professionals who engage in more responsible self-medication practices and who would also promote responsible self-medication practices to their patients, therefore increasing the health literacy of the community overall.

## Figures and Tables

**Table 1:** Characteristics of participants and households (valid N = 60).

	N	%
**Gender**		
Male	18	30.0
Female	42	70.0
**Field of study**		
Medicine	45	75.0
Pharmacy	15	25.0
**Presence of HCPs in household**		
Yes	14	23.3
No	46	76.7
**Presence of children < 14 in household**		
No	41	68.3
Yes	19	31.7

**Table 2:** Main information on drug storage in surveyed households (valid N = 60).

	N	%
**Designated location for drugs (home pharmacy)**		
At a specific place—“home pharmacy”	39	65.0
In several places around the house	20	33.3
There are no drugs in the household	1	1.7
**Availability of drugs to children (<14 years of age)**		
No children in the household	41	68.3
Children in the household—drugs out of reach	19	31.7
Children in the household—drugs within reach	0	0
**Drugs’ shelf-life and purpose**		
There are drugs past their expiry dates	23	38.3
There are drugs of unknown purposes	0	0.0
There are drugs past their expiry dates AND drugs of unknown purposes	2	3.3
No such drugs	34	56.7
Not specified	1	1.7

**Table 3:** Pharmacotherapeutic groups of drugs found in surveyed households (valid N = 54).

Pharmacotherapeutic Groups of Drugs	N	%
Analgesics–antipyretics	50	92.6
Cough and cold products	19	35.2
Antibiotics–chemotherapeutics (overall)	17	31.5
Anxiolytics	9	16.7
Topical dermal drugs, including dermato-antibiotics	8	14.8
Antihistamines	8	14.8
Antihypertensives	7	13.0
Proton-pump inhibitors	7	13.0
Topical ocular drugs, including antibiotics	7	13.0
Vitamins and minerals	6	11.1
Hormones (excl. oral contraceptives)	4	7.4
Other (cumulatively)	17	31.5

## Data Availability

The original contributions presented in this study are included in the article. Further inquiries can be directed at the corresponding author.

## Supplementary Materials

The following supporting information can be downloaded at https://www.mdpi.com/article/10.3390/pharmacy14050116/s1: Supplementary File S1: The Questionnaire.

## Abbreviations

The following abbreviations are used in this manuscript:

HCP	Healthcare Professional
OTC	Over The Counter
ADR	Adverse Drug Reaction
AMR	Antimicrobial Resistance
PPI	Proton-Pump Inhibitor
