# Supplementary material for: Medicine Inventories and Practices of Self-Medication in Households of Medical and Pharmacy Students at the University of Rijeka, Croatia: A Cross-Sectional Study with Implications for Healthcare Professions Education
Source: Pharmacy (Basel). 2026 Aug 7;14(5):116. doi: 10.3390/pharmacy14050116 (PMC13516903; doi:10.3390/pharmacy14050116)
Supplement: Supplementary file 1 [file pharmacy-14-00116-s001.zip › pharmacy-4445315-supplementary.pdf]

Supplementary File S1

Questionnaire (English translation)

1. Field of study
    a. Medicine
    b. Pharmacy

2. Gender
    a. Male
    b. Female

3. Are there any healthcare professionals in your household?
    a. No
    b. Yes – please, select the profession:
        i. Medical doctor
        ii. Doctor of dental medicine
        iii. Doctor of veterinary medicine
        iv. Pharmacist

Please select only one answer for each of the questions below:

4. What is main/predominant source of information on appropriate medicine use in your household?
    a. Independent physician and/or pharmacist
    b. Physician and/or pharmacist who is household member
    c. Product information leaflet within the medicine package
    d. Friends and relatives who are not healthcare professionals

5. Where are medicines stored in your household?
    a. At specific place – home pharmacy/medicine cabinet
    b. Scattered around the house/apartment
    c. There are no medicines in the household

6. Are medicines in your household accessible to children under 14 years of age?
    a. Medicines are kept out of reach of children under 14 years of age
    b. Medicines are kept within the reach of children under 14 years of age
    c. There are no children under 14 years of age in the household

7. Are there any expired medicines or medicines of unknown purpose in your household?
    a. There are expired medicines
    b. There are medicines of unknown purpose
    c. There are both: expired medicines and medicines of unknown purpose
    d. There are neither expired medicines nor medicines of unknown purpose

8. Please, complete the table with information about all medicines found in the household:

Name of the medicine (Brand name/ Generic name)	Dosage	Quantity (number of packages)	Expiry date	Purpose (indication; reason for obtaining that medicine) of the medicine is known (YES / NO)	How often is this medicine used for self-medication by members of the household? (NEVER / SOMETIMES / REGULARLY)
